# Therapeutic Potential of Ursolic Acid in Cancer and Diabetic Neuropathy Diseases

**DOI:** 10.3390/ijms222212162

**Published:** 2021-11-10

**Authors:** Manzar Alam, Sabeeha Ali, Sarfraz Ahmed, Abdelbaset Mohamed Elasbali, Mohd Adnan, Asimul Islam, Md. Imtaiyaz Hassan, Dharmendra Kumar Yadav

**Affiliations:** 1 Centre for Interdisciplinary Research in Basic Sciences, Jamia Millia Islamia, Jamia Nagar, New Delhi 110025, India; manzar987@gmail.com (M.A.); sabeeha.mhg.bhu12@gmail.com (S.A.); aislam@jmi.ac.in (A.I.); mihassan@jmi.ac.in (M.I.H.); 2 Department of Biosciences, Jamia Millia Islamia, Jamia Nagar, New Delhi 110025, India; sarfrazahmed6954@gmail.com; 3 Clinical Laboratory Science, College of Applied Medical Sciences-Qurayyat, Jouf University, Sakaka P.O. Box 2014, Saudi Arabia; 4 Department of Biology, College of Science, University of Hail, Hail P.O. Box 2440, Saudi Arabia; drmohdadnan@gmail.com; 5 College of Pharmacy, Gachon University of Medicine and Science, Hambakmoeiro, Yeonsu-gu, Incheon 21924, Korea

**Keywords:** ursolic acid, clinical trials, diabetic neuropathy, inflammatory diseases, inhibitors, targeted therapy

## Abstract

Ursolic acid (UA) is a pentacyclic triterpenoid frequently found in medicinal herbs and plants, having numerous pharmacological effects. UA and its analogs treat multiple diseases, including cancer, diabetic neuropathy, and inflammatory diseases. UA inhibits cancer proliferation, metastasis, angiogenesis, and induced cell death, scavenging free radicals and triggering numerous anti- and pro-apoptotic proteins. The biochemistry of UA has been examined broadly based on the literature, with alterations frequently having been prepared on positions C-3 (hydroxyl), C12–C13 (double bonds), and C-28 (carboxylic acid), leading to several UA derivatives with increased potency, bioavailability and water solubility. UA could be used as a protective agent to counter neural dysfunction via anti-oxidant and anti-inflammatory effects. It is a potential therapeutic drug implicated in the treatment of cancer and diabetic complications diseases provide novel machinery to the anti-inflammatory properties of UA. The pharmacological efficiency of UA is exhibited by the therapeutic theory of one-drug → several targets → one/multiple diseases. Hence, UA shows promising therapeutic potential for cancer and diabetic neuropathy diseases. This review aims to discuss mechanistic insights into promising beneficial effects of UA. We further explained the pharmacological aspects, clinical trials, and potential limitations of UA for the management of cancer and diabetic neuropathy diseases.

## 1. Introduction

Ursolic acid (UA) is a promising triterpenoid compound present in several plants’ leaves, flowers, and fruits [1]. It shows a broad range of pharmaceutical properties and therapeutic effects [2]. UA has been utilized as a herbal medicine with excellent pharmacological activities [3,4]. It mediates alterations in many signaling pathways and subsequently prevents the progression of chronic diseases [5], demonstrating its anti-inflammatory [6], anti-oxidant [7], anti-carcinogenic [8], anti-diabetic [9], neuroprotective effects [10]. UA is effective in the treatment of many inflammatory diseases [4], including Parkinson’s disease [11] and diabetes [12]. In addition, UA has been implicated in neurodegenerative and psychiatric disorders [13]. It has considerable potential as an oral anti-inflammatory and neural repair drug [14], attenuating neuropathic pain in animal models that can probably be attributed to its anti-oxidant and anti-inflammatory properties [15]. The mechanisms of UA by which applies these beneficial consequences might engage regulation of metabolic and atrophy pathway in skeletal muscles, insulin pathway in adipose tissue, apoptotic and NF-κB pathway in tumor cells, level of oxidants and metabolic pathway in the liver, anti-oxidants and inflammation in the brain [16,17,18].

Cancer is the leading cause of human death in economically developed countries [19,20,21]. The cancer burden keeps on enhancing in developing countries due to increasing aging in the population [21,22]; cancer is a major cause of adult deaths worldwide [21,23]. Cancer is a complex disease that may be inhibited [24,25]; cancer progression might take around 10 to 30 years to develop, starting initiation, promotion for development, and progression [24,25]. Hence, the slow growth of the disease potentially permits interference in the development of tumors into advanced stages and metastases. This disease has a high incidence and mortality rate; its treatment indicates a significant clinical challenge. Hence, the advantages of present chemotherapeutics are inadequate because they tend to cause DNA damage in healthy cells [26,27]. However, targeting the regulation of cancer cells without causing toxic effects in healthy cells is a potential strategy for cancer treatment [28,29].

Diabetes mellitus (DM) is a chronic metabolic disorder linked with the emission and sensitivity of insulin [30]. Based on pathogenic developments which cause hyperglycemia, DM is categorized as type 1 or type 2 [31,32,33]. The specific mechanisms to insulin resistance development are still not completely elucidated. A low-grade chronic inflammation, uptake of glucose in adipose and skeletal tissues and various flaws in the intracellular insulin pathway play crucial functions in insulin resistance [34]. Diabetes is a significant health problem, comprising a complex disorder strongly related to nephropathy, cardiomyopathy, and neurodegeneration [35]. Diabetic individuals might suffer from peripheral and central nervous neuropathy, which is linked with impairments in memory and learning [36,37,38,39,40]. 

Diabetic neuropathy (DN) is a common and challenging complication of DM, leading to the most morbidity and mortality, resulting in a vast economic burden to diabetes care [41,42,43]. Neuropathy is generally widespread; distal symmetric polyneuropathy is very general, leading to sensory disturbances [42,43,44,45]. DN is a failure of sensory function starting distally in the lower extremities, which is considered via pain and substantial morbidity. Hence, 50% of individuals with DM develop DN [46]. DN is a specific neurodegenerative disease of the peripheral nervous system, which targets autonomic and sensory axons at later stages to motor axons with a lesser degree [46]. DN is induced by hyperglycemia and dyslipidemia that modify the insulin signaling pathway, which causes numerous pathological modifications in vascular cells, glia and neurons, leading to dysfunction of nerve and ultimately neuropathy [46,47].

Inflammation engages the activation and employment of phagocytes (neutrophils, macrophages), NK cells, and the discharge of cytokines through activated cells that are crucial to the host defense system. Since the chronic inflammation that continues yet after removal of the pathogen(s) was linked with many diseases, including cancer [48], neoplasms, inflammatory bowel disease [49], Alzheimer’s disease [50], therefore, dysregulation of enzymes and cytokines, might contribute to the pathogenesis of several chronic inflammatory diseases [51]. NF-κB, NF-AT, and AP1 with JNK, ERK, and p38 are well-known for regulating inflammatory cytokines and enzymes targeted through various investigators to improve chronic inflammation [52,53,54]. Inflammation plays a fundamental role in the pathogenesis of DN [55].

The complexities of DN and inflammatory diseases have considerably evolved over the years. Efforts for understanding the etiology of DN and inflammatory diseases are in progress to design attractive therapeutic approaches. In this context, UA may be a potent therapeutic molecule for DN and inflammatory diseases. UA and its derivatives are potential therapeutic candidates that can be explored to treat DN and inflammatory diseases. In this review, we systematically collected current findings concerning biochemistry, potency, pharmacological aspect, drug delivery system, and clinical trials of UA.

## 2. Structure and Biochemistry of UA

A pentacyclic triterpenoid; UA (3β-3-hydroxy-urs-12-ene-28-oic-acid), contained a C-30 chemical structure made from units of isoprenoid through the A, B, C, D, and E rings [56], with a molecular mass of 456.71 g/mol and has the chemical formula of C_30_H_48_O_3_ [57]. Triterpenoids are secondary metabolites, including more than 20,000 reported compounds linked with many biological mechanism activities [58]. UA was first documented in 1920 [59]. It is soluble in hot glacial acetic acid and alcoholic sodium hydroxide [57]. UA is generally biosynthesized from dammarenyl cations by folding and cyclizing squalene, making the fifth ring of UA via a ring addition, with three oxygen atoms in the transition metal atom [57]. UA biosynthesis found in plant cells derives from cyclical (3S)-oxidosqualene cycling. Hence, the pre-dominant 3S predecessor is changed into a cationic dammarenyl construction undergoing chain growth, making this third separate ring present in the amyrin skeleton, a nucleus in UA [60]. Table 1 illustrates the UA sources with their biological properties.

UA’s limited bioavailability, solubility, and quick metabolism have hindered their extra clinical functions to therapeutic consequences in various diseases. However, several researchers have identified several alterations to improve UA potency, bioavailability, and solubility in water [61,62]. Many attempts have been completed to synthesize chemical imitation of UA to enhance its efficacy, therapeutic potential and solubility [63,64,65]. The basic construction of UA has been observed in the serum of mice following oral administration and was found to be absorbed well in the gastrointestinal (GI) tract [66]. However, UA was observed in the rat’s cerebrum, 1 h after the oral administration. This suggests the ability of UA in crossing the blood-brain barrier (BBB) [67]. Furthermore, for improving its absorption, UA was administered in liposomes during clinical trials [68,69]. Hence, several preclinical, and clinical trials using UA, indicate promising effects for neurodegenerative and psychiatric diseases [13].

## 3. Biological Potency of UA

The molecular action of bioactive molecules might open up novel opportunities to the scientific community for developing and improving new therapeutic approaches for tackling dreadful diseases, including neurodegenerative disorders. UA is one such plant-based therapeutic metabolite, which plays a vital function in cell death, angiogenesis, metastasis, and inflammatory processes [59]. Table 2 presents various health benefits, including anti-apoptotic, anti-oxidant, anti-inflammatory, anti-carcinogenic, anti-rheumatic, anti-tumoral, anti-viral, trypanocidal, etc., of UA [81,82]. UA has anti-cancer activity due to low toxicity and commercial accessibility forms [80,83].

UA is popular due to its anti-proliferative properties, the incentive of tumor cell death, an obstacle of tumorigenesis, and inhibiting the cell cycle in tumors cells. Hence, examining one cell death mechanism that indicated UA could prevent NF-κB pathway through p65 phosphorylation repression, effecting a mandatory reduction in several downstream oncogenes, including Bcl-xL and Bcl-2. The anti-cancer effectiveness of UA is weak due to its lesser solubility that reduces the drug absorption in the human body system, which causes challenges in achieving comprehensive benefits. However, it is required for making its derivatives by semi-synthetic alterations for improving its anti-cancer action. Frequently, the modifications of UA occur on positions C-3, C12–C13, and positions C-28 [63,76,95]. Antimicrobial activity of UA compounds against *Mycobacterium tuberculosis* H37Rv has been recently reported [96,97]. UA is currently in different phases of clinical studies due to its therapeutic effects and selectivity against several diseases [98,99].

## 4. Bioavailability and Pharmacokinetic Properties of UA

Cell membrane permeability and pharmacokinetics are vital in the clinical development and improvement of novel biologically active agents/compounds with an outlook for considering their performance in vivo and establishing the most acceptable dosage regimen. Hence, pentacyclic triterpenoids usually suffer from less oral bioavailability [100]. In the organism, a vast inter and intra character absorption variability shows challenges in accomplishing secure and efficient concentration of drug [101]. Physicochemical characters of the molecules are the first reason which affects bioavailability [102]. UA is a low molecular weight compound [58], with three hydrogen bond donors and acceptors. However, these properties for estimating the drug-likeness are in harmony with Lipinski’s rule [103]. UA has more lipophilicity [100] and less wettability [104]. Hence, their absorption is obstructed through slow partitioning between extracellular fluid and cell membrane and poor dissolution. UA can be inserted in the phospholipid bilayer while not taken up via cells [104].

Moreover, aqueous solubility is exaggerated through the crystalline structure of natural UA. Decreased particle size and amorphous state remarkably increased rate of dissolution and solubility of triterpenoid [105]. Adverse characteristics of UA should be addressed in the improvement of pharmaceutical dose shapes. Hence, for overcoming biological barriers, the second main factor is the capability of the drug [102]. The proof from in vitro permeability investigations involves which passive diffusion is the primary process of UA transport [102]. Hence, apparent permeability coefficients, estimated applying Caco-2 monolayers, have been in limits of oral absorption [102], which instant glucuronidation, and sulfation in the intestinal cells, are extremely unlikely [106]. UA is a substrate of cytochrome P450 and P-glycoprotein. Therefore their bio-availability can be limited through biotransformation and active efflux [105,107]. 

Many pharmacokinetic examinations have exposed where maximal plasma concentration subsequent oral administration of doses up to 300 mg/kg has been low, and removal half-life has been comparatively short (<1 h) [69,105]. This pharmacokinetic outline shows that fast elimination and tissue distribution in the pharmacological results of UA may not be honestly related to plasma concentrations. UA has broad tissue distribution, such as in the animal’s testes, colon, lung, kidney, spleen, brain, liver, heart and bladder [69,108]. The liver is the main organ of the triterpene disposition [108], and liver-related doses limit the toxicity in clinical trial phase I of UA liposomes [109]. UA can cross the blood-brain barrier, and they have potent neuroprotective effects [108]. Targeted delivery for the brain may be attained through particular delivery systems that use UA nano-lipid vesicles in the form of intranasal gel [110]. Hence, high lipophilicity influences triterpenoids for liver metabolism. However, a tissue distribution examination in mice has shown that the UA concentration in plasma was gradually reduced, but the concentration in the liver increased [111].

## 5. Pharmacological Aspects of UA

### 5.1. Anti-Diabetic Effect of UA

DM is a chronic disease, which is regulated via deficiency or insulin resistance. DM is one of the main risks for humans globally [103]. Long-term utilization of hypoglycemic agents might decrease their pharmacological action. Due to a complete glucose insufficiency, type 1 diabetes mellitus (DM1) affects hyperglycemia. Hence, this affects various macro and microvascular alterations in pathology, osteopenia, neuropathy, DN, diabetes, and osteoporosis. However, clinical trials have indicated that DM1 enhances tibia, vertebral, proximal humerus and knee rupture risks independent of bone mineral density (BMD) [86]. In diabetic mice, UA is well-recognized to impart beneficial effects in lowering the concentration of blood glucose and curative of related diabetic problems. Hence, UA decreases protein tyrosine phosphatase 1B, gets better phosphorylation of insulin receptors that induce glucose absorption [112].

Obesity and diabetes require inhibition of the pancreatic α-amylase for the treatment because it decreases blood glucose levels in vitro and in vivo [84,113]. A recent study has estimated the UA effects on body weight and glucose tolerance in the metabolic syndrome samples, who obtained UA 150 mg before breakfast to 12–20 weeks [87]. Decreases in blood glucose level, mass index and body weight have been detected in patient samples, suggesting it considerably develops insulin sensitivity. Hence, Chu et al. [114] 0.5% UA diet affected a reduction in body weight and free fatty acids by uncoupling protein 3/AMPK dependent signaling in the HFD mediated obese rats after 06 weeks of the treatment. Reduction in glucose intolerance and body weight gain was observed in mice treated with 0.14% UA diet for up to 6 weeks [115]. Li et al. [113] reported that, in HFD induced obese mice, UA reduced insulin resistance and body weight gain. Hence, 80 μM UA decreases cholesterol and triglyceride levels by reducing the synthesis of fatty acid and enhancing fatty acid oxidation in the hepatocytes, which up-regulation of PPAR-α expression is probably vital to the useful effect of UA [116].

Treatment with UA (50 and 200 mg/kg) reduces lipid accumulation, TG levels and body weight. It enhances HDL cholesterol, insulin sensitivity, brown adipose tissue, and β-oxidation in the HFD-mediated obese rats, which improves energy expenditure [117]. Another exciting study indicated that UA (0.05%) blocks insulin resistance and glucose intolerance through conserving pancreatic β-cells in diabetic mice [85]. Since UA effects were identified by Kunkel et al. [115], UA is the primary controller of glucose levels in diabetes [84]. The AMPK regulation levels through blocking liver enzyme B1 are critical to the treatment of diabetes and obesity. Hence, this suggests the significance of UA in diabetes treatment. The anti-diabetes effect of UA is described in Table 2.

TGR5 activation via a chemical agonist might enhance incretin secretion and decrease the blood sugar level. UA has the potential effect on TGR5. UA concentration enhanced glucose uptake in the CHO-K1 cells, which express TGR5. TGR5 may improve cAMP for activating PKA and downstream pathways. UA stimulated a concentration-dependent increase in GLP-1 emission blocked through triamterene on the efficient concentrations for blocking TGR5 in NCI-H716 cells [118,119]. TGR5 agonist was positive results in T2DM-like animals [120]. UA enhanced the plasma GLP-1 level by activating TGR5 that has been illustrated in vivo with type 1-like diabetic rats. UA may trigger TGR5, increasing GLP-1 secretion in vitro as well as in vivo. However, UA is appropriate to use in the activation of TGR5 [118].

### 5.2. Neuroprotective Effect of UA

Neurological disorders comprise anxiety, stroke, depression, PD, and AD [76,121,122,123,124]. Long-term neurological effects may not be developed through subarachnoid hemorrhage (SAH) therapies, which decrease the occurrence of cerebral vasospasm, showing their significance in the patient result has been misconstrued. Early brain injury (EBI) was observed as the primary reason for adverse effects in SAH patients.

**Figure 1:**
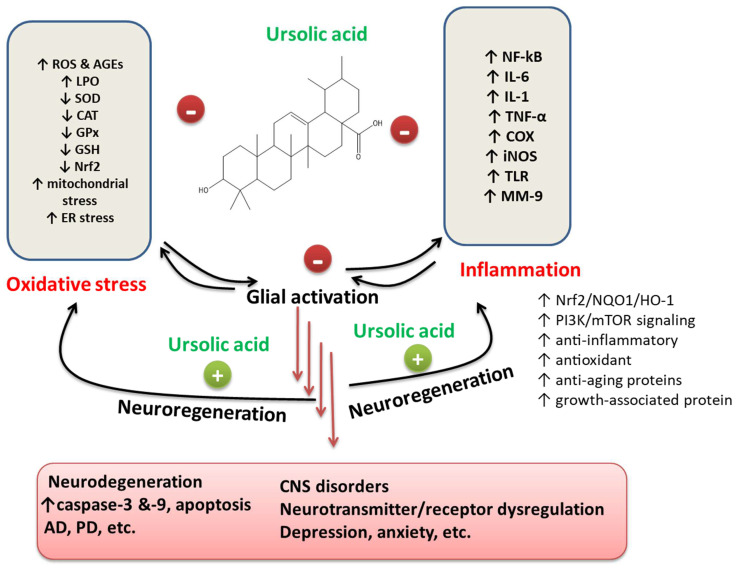
Anti-inflammatory and anti-oxidant mechanisms of neuronal function and neuroprotection through UA. Glial cells play a critical function in neuro-inflammation and oxidative stress, common in various neurodegenerative diseases. By repressing the making of ROS, AGEs, and LPO products and enhancing anti-oxidant defenses by upregulation of the Nrf2 signaling, UA shows neuroprotective consequences in neuronal cells (Habtemariam S, 2019). UA has various roles in the CNS. (−), inhibition; (+), promotion; ↓, Decrease; ↑, Increase.

Several reports suggest UA remains a vital and admired anti-oxidant reagent; for example, it has a brain defensive effect for ischemic stroke [88]. Oxidative stress was reported as being amongst the complicated reasons linked with neuronal death following SAH. ROS and H_2_O_2_ play a critical function after SAH [125]. Redox-sensitive Nrf2 activation has a central location in getting a better endogenous protection process by which the brain defends itself against ischemic injury and improves from the stroke [126]. Pro-inflammatory and oxidative actions are involved in many CNS-related diseases (Figure 1). The neuroprotective results of UA against a model of ischemia [126]. The results of UA in decreasing EBI might be described through mitigation of oxidative stress at SD rats. Hence, UA may be considered a potential therapeutic drug for neurological diseases [88].

PD is a chronic progressive neurodegenerative disease described by motor and non-motor features [127,128,129,130]. Its increasing degenerative effects on muscle regulation and mobility have a key clinical result for caregivers, families and patients [131]. Hence, in age-related developing neurodegenerative disorders, following Alzheimer’s, it is the second most frequent and is characterized via dopaminergic (DA) neuron decrease and neuroinflammation [89,132,133]. The neuroprotective efficacy of UA in 1-methyl-4-phenyl-1,2,3,6-tetrahydropyridine (MPTP)-mediated PD mouse models. Hence, the authors identified; UA enhances cognitive deficits, repairs altered dopamine levels, and defends the MPTP intoxicated mouse’s DA neurons. Along with different doses, the main competent dose to PD was 25 mg/kg body wt. [89].

**Figure 2:**
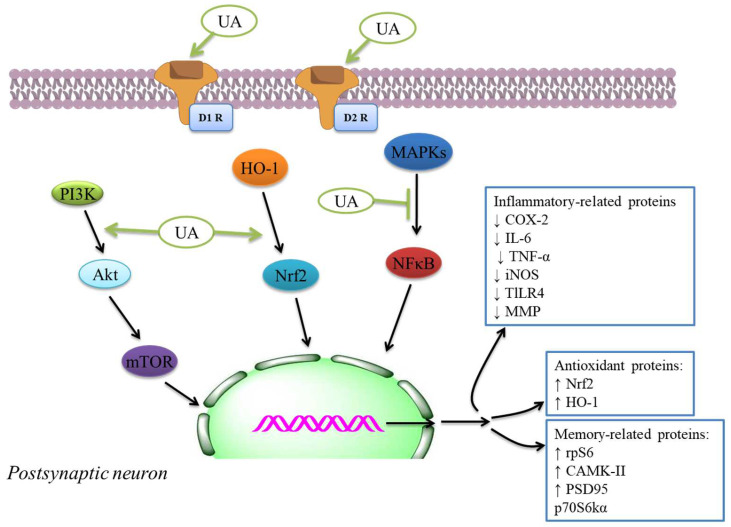
Descriptive proposal of the molecular targets altered through UA in a putative synapse. UA can modulate various receptors, including dopamine D1 and D2. UA might trigger nuclear factor (erythroid-derived 2)-like 2/heme oxygenase 1 (Nrf2/HO-1) and PI3K/Akt/mTOR, that can inhibit MAPK/NF-κB-mediated pathways. These intracellular pathways’ inflection can provide the expression of proteins involved in cognitive improvement and anti-oxidant and anti-inflammatory effects. D1R, dopamine receptor 1; D2R, dopamine receptor 2; ↓, Decrease; ↑, Increase.

Oxidative stress and excitotoxicity are the two mechanisms that have been continually demonstrated as being involved in a broad range of diseases of the brain and nervous system [134,135,136,137,138,139,140]. It can lead to stable brain harm and reduction of cognitive functions [141,142,143]. The first investigation focusing on the protective consequence of UA on neurons was performed by Shih et al. [144]. UA significantly reduced and repressed free radical production. The study [145] was examined the protective result of UA against galactose-mediated damage in mice brains. Repressing NF-κB through UA as a process for attenuating cognitive deficits and avoiding brain damage was identified by Wang et al. [146] and Li et al. [126]. These models have been lipopolysaccharide-damaged mouse brains after cerebral ischemia [93], illustrating intracellular pathways engaged in UA neuroprotective activity. Hence, UA may trigger PI3K/Akt pathway and repress FoxO1 activity in domoic acid-mediated mice. PI3K/Akt activation appears to motivate UA’s results in the excitotoxicity model [93]. UA was capable of restoring the levels of knowledge and memory-connected proteins, including postsynaptic density protein 95 and Ca^2+^ calmodulin-dependent protein kinase II mediated via a high-fat diet through activating the mTOR pathway induced via p70 S6 kinase alpha and S6 ribosomal protein in the hippocampus [147]. These results might induce the activation of PI3K and mTOR pathways (Figure 2). Hence, the neuroprotective effects of UA have been examined against the neurochemical and behavioral modifications induced through MPTP for model PD [89].

Oxidative stress and excitotoxicity usually result in mild to severe nervous system defects [2]. Inequity in the cellular homeostasis might everlastingly decrease cognitive function and affect brain injure [141,148], resulting in a variety of brain diseases [149]. Hence, the effects of UA on brain disorders that block oxidative stress [88] and excitotoxicity [144] suggest; it can play a protective function in several brain disorders induced through excitotoxicity and oxidative stress. However, UA represses the apoptotic pathway [88] and wields anti-inflammatory results in the brain [91]. Researchers have identified that UA blocks the activity of MMP-9, which is a potent cause of several tumors in C6 glioma cells [91,150]. UA might repress NF-kB-dependent signaling pathways, which are triggered via TNF-α/IL-1β [151]. In a rat model of cerebral ischemia and reperfusion injury, Wang et al. [90], showed the relationship between UA and MMP-2/-9. Since the activities of MMP-2, and MMP-9, were repressed through UA administration. Hence, UA has a defensive effect against several inflammatory situations of the brain.

In cerebral ischemia and reperfusion injury, TLRs play a critical role by inducing the making of inflammatory mediators, including ILs and TNF-α [152,153,154]. TLR4 was primarily reported as receptors to endogenous ligands such as DAMPs, and HMGB1, during brain injury. UA controls the TLR pathway and shows prominent anti-inflammatory functions. UA demonstrates biological actions in the brain, such as anti-inflammatory, anti-oxidative, anti-rheumatic and anti-tumor effects [155]. UA decreases inflammatory cytokine-making to protect the brain from cerebral ischemia and reperfusion injury, probably by HMGB1/TLR4/NF-κB pathway [156]. UA might be helpful as a potential efficient adjunct for therapy to ischemic brain injury before reperfusion.

### 5.3. Anti-Inflammatory Effect of UA

Inflammation plays an essential role in developing and progressing multiple diseases, including neuropathy, insulin resistance, and diabetes [157], cancer [158,159,160,161,162]. After injury and microbial attack, the inflammatory reaction is started to recover homeostatic tissue equilibrium between composition and physiological role. Hence, persistent inflammation can cause harm to tissues, affecting non-functioning tissues/organs [163,164]. Inflammation is an obscured incidence connected to the progression of various diseases, including neurodegenerative diseases and cancer [165]. Acute inflammation, with attendant cytokine action and the increased output of ROS, is reported like a tumor-promoting disease [166,167,168]. For detecting the anti-inflammatory activity, the study reported UA’s capability to reduce the making of TNF-α in A549 and RAW 267.4 cell lines infected by *Mycobacterium tuberculosis* and Con A-stimulated mouse splenocytes.

The authors examined UA action intending to reduce COX-2 and NO synthase levels found in roused cells. Hence, UA showed a considerable inhibitory effect of cytokine levels, immunomodulatory mediators, and liberate of NO. This compound may be used for tuberculosis and antibiotic therapy due to the UA’s anti-inflammatory effectiveness in cells [169]. The study showed in vitro inhibition of COX-2 action was because of UA and cranberry extracts [170], indicating potential anti-inflammatory and anti-oxidant activity (Table 2). The anti-inflammatory activity was investigated by utilizing enzyme inhibitory examine in vitro COX-1 and COX-2 [171,172]. The molecular docking discoveries exhibited; the UA derivatives indicated elevated attraction to effective COX-2 site, potentially showing anti-inflammatory effectiveness by COX-2 inhibition. Hence, UA and its derivatives showed anti-inflammatory activity that might cause the development and improvement of potentially novel and secure COX-2 inhibitors [173,174]. The anti-inflammatory mechanisms inhibit main inflammatory cytokines, iNOS and COX expressions, and anti-oxidant mechanisms like the activation of Nrf2 signaling. The inflammatory and anti-oxidant mechanisms of neuroprotection through UA [175] are also indicated by the plethora of extra systemic results of UA in several experimental models [175]. The effects were linked with the inhibition of NF-κB translocation to the nucleus (Figure 2) and decreased expression of iNOS, COX-2, TNF-α, and interleukin-6 that decreased the phosphorylation of p38MAPK in the mouse brain [146].

Terpenoids could have anti-oxidant results in vivo through mediating anti-oxidant defenses, including CAT, SOD, GPx, and GR, exhibited to their therapeutic potent in AD by a range of assays [176,177,178,179,180]. These compounds display anti-oxidant effects and possess anti-inflammatory functions. They are tackling complex diseases by what has been explained as one drug → multi-targets → one/many disease(s) therapeutic theory [181]. In this perspective, the therapeutic promising of UA like a prototype lead is exhibited in several CNS diseases, mainly by anti-inflammatory and anti-oxidant mechanisms. The anti-oxidant–anti-inflammatory axis was indicated to play a function in the anti-diabetic effect of UA as displayed in streptozotocin-mediated rats [182] in db/db diabetic mouse model [183], DN models [184], diabetic-mediated monocyte dysfunction in mice [185], aortic damage in the STZ-mediated diabetic rats [186].

### 5.4. Anti-Cancer Effect of UA

Cancer is a complex disease due to tumor heterogeneity, a major limitation of chemoprevention [187,188,189,190,191,192]. The anti-cancer potency of these agents was analyzed in several tumor cells, like, A-549, THP-1, HCT-116, MCF-7, and ordinary adult epithelial cells (FR-2). The anti-cancer potencies of UA and its derivatives against these cells were detected [193]. UA caused Bcl-2 down-regulation and Bax up-regulation and released cyt-*c* from mitochondria to the cytosol. Hence, UA cleaved caspase-9 and decreased mitochondrial membrane ability. The mechanism of UA is dependent on the death receptor. UA might be used as a promising substance of biological source while it comes to cancer prevention and therapy, changing their metabolism to prevent metastasis and angiogenesis [2,194,195].

Researchers have examined the valuable results of UA on cell metabolism in humans and rodents. Hence, the mechanisms fundamental to the anti-tumor development of UA [196] and tumor cell proliferation [197], inflection of cell death [198], inhibition of cell cycle arrest [199]. UA was showed useful effects on cell death and autophagy in breast cancer cells. Lewinska et al. [198] identified that 20 μM UA blocks the activation of Akt and promotes apoptosis and autophagy in cancer cells. Hence, it also reduces the pERK1/2 level. UA provokes Akt activation, enhances the oxidative system, and reduces the ATP levels, lactate, and glycolytic enzymes, including pyruvate kinase and hexokinase 2 in breast cancer cells [200,201]. It reduces ATP making and activates AMPK that inhibits proliferation in cancer cells [202]. UA can be a potential controller of AMPK that blocks glycolysis and cancer survival in vivo [201]. It inhibits cell proliferation in tumor cells [203]. Yan et al. [204] identified UA activates a pro-apoptotic pathway in liver tumor cells. UA exerts considerably improved pro-apoptotic results through enhancing the caspase-3 and caspase-8 levels and DNA fragmentation in tumor cells. Hence, UA reduces Na+–K+-ATPase action and MMP, showing mitochondrial dysfunction in cancer cells [16].

Autophagy facilitates the continuance of metabolic precursors regeneration and cellular homeostasis. The function of autophagy in cancer might be viewed as a controversial procedure. The process of autophagy may be a selective as well as a non-selective mechanism. Some studies showed that autophagy inhibits cancer progression and development [205], and on the other hand, studies indicated that autophagy has a protective function on cancer cells [206]. UA stimulates autophagy in A549 cells [207]. UA-mediated autophagy in PC3 cells is linked with decreased cell viability [208]. However, UA activates the mTOR/Akt pathway and through the modulation of PI3K/Akt pathway, UA induces autophagy [208]. In response to growth factors and nutrient accessibility, autophagy is triggered via mTOR/PI3K/Akt, which controls protein synthesis and cell growth [209].

## 6. Drug Delivery System

UA and its derivatives have been involved in various pharmacological applications linked with the prevention of diseases. UA can be used as a potential applicant for developing a comprehensive capable strategy towards preventing and treating health disorders [59]. UA displays a variety of pharmacological effects, less solubility in the aqueous medium that affects its therapeutic function and bioavailability because the polarity and solubility of the material may affect its capability for penetrating biological membranes [210]. Several strategies might be utilized for overcoming these limitations, including particle size reduction, chemical alterations, salt creation of the molecules, application of surfactants, pH adjustment of agent/drug into various delivery systems [211,212]. However, drug delivery systems were exploited with significant achievements for improving UA’s Physicochemical functions and therapeutic application. Besides improving solubility, they also change drug discharge and enhance the bio-availability of hydrophobic agents and drugs [213]. Various nano-carriers have been utilized for UA delivery, including nanoemulsions, polymeric nanoparticles, mesoporous silica nanoparticles, liposomes, solid lipid nanoparticles, and solid dispersions [58].

The study’s pH-sensitive mesoporous silica nanoparticles have been established to be biocompatible and permitted sustained liberation of UA [214]. Moreover, that is increasing the cytotoxic result against HepG2 cells contrasted with free UA. Another study that examined the development in drug dissolution profile [215] estimated the in vitro dissolution summary, trypanocidal effect, and cytotoxicity effect of UA by the nanoemulsions-based delivery system. Nahak et al. [216] reported the loss in UA crystallinity after analyzing the UA loaded in solid–lipid nanoparticles physicochemical characteristics. 

The lipid matrix influences the rate of drug discharge and agent/drug discharge patterns, showing an anti-cancer effect more significant than free UA against K562 and B16 cells. Long-circulating and pH SpHL-UA were utilized to improve drug administration intravenously, its anti-tumoral result on LNCaP and MDA-MB-231 tumor cells [217]. Hence, solid dispersions may be an attractive approach for the delivery of UA. These contain molecular combinations of carriers with hydrophobic agents/drugs, which are amphiphilic/hydrophilic, which permit a decrease of drug particle size that may develop the solubility and modulate drug discharge profile [218]. Hence, some new technologies have been applied for producing UA preparations, which may modify the pharmacokinetics procedure and enhance its solubility and bioavailability [219].

## 7. Preclinical and Clinical Studies of UA

### 7.1. In Vitro Studies

Extensive studies have produced data representing the in vitro [220] and in vivo [197] anti-tumoral effects of UA [221], identifying its capability for adapting molecular targets engaged in the progression and development of psychiatric and neurodegenerative disorders. However, the capability of UA to apply neuroprotective results against excitotoxicity, a crucial process linked in neurodegenerative disorders, including PD and AD, might be related to its probable therapeutic potency in the management of diseases [13,222]. A broadly applied in vitro PD model contains mediating excitotoxicity for neurons with MPTP, its dynamic metabolite. Since neurotoxic compounds are likely to accumulate in DA neurons, they induce ROS and stimulate oxidative imbalance and inflammatory damage [223]. The neuroprotective result of UA against MPTP has been identified in PC12 cells [224]. AD, one of the largest established progressive neurodegenerative diseases [225], is generally distinguished through cognitive impairment and progressive neurodegeneration [226]. Hence, one of the major neurochemical modifications for AD is making amyloid plaques enclosing protein fragments called Ab [227]. The Ab plaques can stimulate neurotoxic effects via enhancing ROS manufacture, inflammation, and cell death [227].

The participation of inflammatory processes in the progression and development of AD was broadly identified [228]. In particular, enhanced making of cytokines and ROS and neuroinflammation can result from Ab accumulation [228]. Activating MAPKs starts a signaling pathway, which leads to the activation and translocation of NF-κB to the nucleus, which is involved in making and liberating pro-inflammatory molecules [229]. UA can also show defensive activity against an Ab-mediated neuroinflammatory result [230,231]. Treatment of PC12 cell lines by UA inhibited the expression of iNOS and COX-2-mediated through Ab_25–35_ [231]. This anti-inflammatory result of UA is induced, at any rate partially, via the decline in the phosphorylation level of ERK1/2, c-Jun NH2-protein kinase, and p38MAPK that subsequently can block NF-κB translocation to the nucleus [231].

### 7.2. Clinical Trials

The final aim of cancer and neurological research is the functioning and implementation of drugs or compounds for clinical utilization. Presently, UA is undergoing phase I studies for evaluating its safety and adverse effects in the patients. Hence, due to less water solubility and low bioavailability of UA was administered as liposomes. Studies have been reported [109,232,233], UA liposomes exhibited adequate toxicity and adverse effects. The most common objections were diarrhea, nausea, and skin troubles. The conclusion of these studies and analysis was the requirement of the continuance of investigates during phase II studies. The combination of UA sub-effective doses with bupropion, fluoxetine used a synergic anti-depressant effect in TST [234,235]. These effects recommend; UA treatment might improve the efficiency of anti-depressants. Monoaminergic neurotransmission may be concerned with the anti-immobility results of UA in TST. This mechanism of action is a characteristic of triple reuptake anti-depressant agents or compounds [236] that act via blocking the reuptake of NE, 5-hydroxytryptamine, and DA. This group of drugs comprises a novel anti-depressant plan and strategy, affording a more quick response outline, an improved efficiency, and fewer side effects compared with particular or double monoamine receive inhibitors in the clinical studies [237,238,239]. Hence, UA administration can be a potential anti-depressant strategy.

A recent study has estimated the new formulation in 63 Chinese persons, including 4 advanced solid tumor patients and 59 normal samples. In the single administration by oral UANL (37, 74, 98, and 130 mg/m^2^), the high concentration of UA in plasma was attained in 4 h [240]. One clinical trial calculated the result of UA after 8 weeks of resistant exercise in the male samples [241]. In this examination, 16 normal men were given three capsules of UA (450 mg) every day for 8 weeks [241]. The subjects were given UA and decreased their percentage of body fat and enhanced muscle potency without modifications in skeletal muscle mass [241]. In addition, the blood levels of irisin and insulin-like growth factor-1 were enhanced in the subjects. Improvements can be associated with the UA effects in skeletal muscle [241]. Many studies were indicated that the improved making of irisin in the muscle induced through physical exercise could encourage the making of neurotrophins in the hippocampus [242], neurogenesis, and synaptogenesis in rodents [243]. Multiple synthesized methods based on UA with anti-diabetic, anti-inflammatory, and cardioprotective were identified [243], the neuroprotective potential of UA and analogous compounds, advising, which they can be helpful to the management of PD and AD.

## 8. Conclusions and Future Directions

UA is a natural compound, which humans have securely utilized in different forms. UA shows various biological effects and exhibits its potential effects in the prevention and treatment of several pathologies. Hence, the pharmacological results of UA showed anti-cancer, anti-diabetic, neuroprotective, and anti-inflammatory mechanisms in the cellular and animal models. A new potential opportunity could be managing cancer, diabetic neuropathy, and inflammatory diseases with UA based on its pharmacological functions. UA’s anti-cancer and neuroprotective activity might be linked with its capability to stimulate the anti-oxidant defense systems and down-regulating the pro-inflammatory signaling pathways in the neurodegeneration and ischemic brain alter models in vitro and in vivo. Several in vitro and in vivo studies identified that UA is a promising phytocompound in treating and managing cancer. The beneficial role of UA to neurodegenerative diseases is that it allocates several neurobiological mechanisms, including oxidative stress, neuroplasticity, neuro-inflammation, and impaired signaling pathways. 

In addition, preclinical trials have recommended the beneficial effects of UA on traumatic brain injury, cerebral ischemia, and brain hemorrhage. Hence, all these potential preclinical trial studies, merely a limited number of clinical trial studies, have examined the promising beneficial results of UA to the prevention and treatment of cancer and diabetic neuropathy diseases. Hence, UA appears to be a potential therapeutic strategy that justifies prospective clinical trial studies, believing it’s safe to utilize. Preclinical and well-conducted clinical studies have modified practice parameters and more personalized moves toward treating diabetic neuropathy and inflammatory diseases. Our perceptive of clinical studies presentation and best possible therapeutic management of cancer and diabetic neuropathy from the establishment to current paradigm modify in preclinical trials research space.

Accumulating clinical and preclinical studies research in the subsequent decade may and will revolutionize this scenario. Further investigations and applications might facilitate the opportunity for UA to be improved and developed in new drugs to treat several human diseases, including cancer and diabetic neuropathy. In addition, the cellular and molecular mechanisms’ most important effects of UA in cancer and diabetic neuropathy diseases should be further investigated for implementing UA as a workout mimetic.

## Figures and Tables

**Table 1:** Sources of UA with their biological properties.

Plants Species (Family)	Vegetal Part	UA Content (mg or g)	Type of Study	Biological Effects	References
*Eucalyptus obliqua* (Myrtaceae)	leaves	nr	in vivo	Neuroprotective agent	[70,71]
*Malus pumila* (Rosaceae)	fruits	nr	in vitro	Anti-tumor	[72]
*Tribulus arabicus* (Zygophyllaceae)	aerial parts	1 g	in vitro and in vivo	Anti-hyperuricemic activity	[73]
*Panax ginseng* (Araliaceae)	roots	nr	in vivo	Anti-hyperlipidemic and anti-oxidant effects	[74]
*Bursera cuneata* (Burseraceae)	aerial parts	33.3 mg	in vitro and in vivo	Anti-inflammatory and anti-histaminic activity	[75]
*Sambucus australis* (Adoxaceae)	aerial parts	180 mg	in vitro	Antibacterial and anti-oxidant	[76,77]
*Fragrae fragrans* (Gentianaceae)	fruits	91 g	in vitro	Anti-proliferation	[78]
*Saurauja roxburghii* (Actinidiaceae)	leaves	nr	in vitro	Cytotoxicity against glioma cells	[79]
*Ocimum sanctum* (Lamiaceae)	whole plant	11.21 mg	in vitro	Anticancer and anti-proliferation	[80]

nr = not reported.

**Table 2:** UA effects on diabetes and brain pathologies.

Disease	Experimental Subject	Dosage	Beneficial Effects	References
Diabetes	3T3-L1 adipocytes	1 μg/mL for 10 min	↑ Akt, insulin receptor, and GLUT 4 ↑ Glycogen synthase kinase-3β	[84]
Diabetes	Streptozotocin-injected male ICR mice	0.5 g/kg for 4 weeks	↓ TNF-α and Glucose ↑ Insulin (pancreatic, plasma)	[85]
Diabetes	Streptozotocin-injected male mice	200 mg/kg per day for 6 weeks	↓ Adipocyte dysfunction ↓ Fasting blood glucose ↓ PPAR γ and aP2 ↑ Bone formation	[86]
Metabolic syndrome	Diagnostics of metabolic syndrome patients	Orally 150 mg/kg for 12 weeks	↓ Body weight, BMI, and waist circumference ↓ Fasting glucose	[87]
Subarachnoid hemorrhage (SAH)	Male Sprague Dawley experimental SAH rat model	25 and 50 mg/kg at 0.5, 24, and 47 h after SAH	↓ MDA ↑ Neurological score ↑ Cerebral vasospasm ↓ BBB permeability (EB content) ↑ GSH/GSSH ratio, SOD activity, and Catalase activity ↓ Apoptotic index ↓ Caspase-3, -9 mRNA expression	[88]
Parkinson’s disease	Male Swiss albino mice	5, 25, and 50 mg/kg for 21 days	↑ Rotarod test ↑ Hanging time ↓ Nitrite level ↓ Narrow beam walking test ↑Acidhomovanilic acid ↑ Dopamine	[89]
Cerebral ischemia and reperfusion injury	Male Sprague Dawley rats	5, 10, and 20 mg/kg at 0.5, 24, and 47 h after reperfusion	↓ Neurological deficit score ↓ Infarct volume ↑ PPARγ protein level ↑ Number of intact neurons ↓MMP-2 & -9 protein levels	[90]
IL-1β or TNF-α- induced C6 glioma invasion	Rat C6 glioma cells	5, 10, and 20 μM for 24 h	↓ MMP-9 activity by TNF-α or IL-1β ↓ IκB kinase activity by IL-1β or TNF-α ↓ IκBα activity by IL-1β or TNF-α ↓ NF-κB activity	[91]
D-Galactose-induced neurodegenerative changes	Male Kunming strain mice	10 mg/kg for 8 weeks	↓ ROS level ↓ AGEs level ↓ Number of CD11b-stained cells, ↓ Carbonyl protein level GFAPstained cells, and RAGE- positive cells ↓ iNOS, IL-6, IL-1β, COX-2, and TNF-α protein levels	[92]
Domoic acid-induced cognitive deficits	Male ICR mice	100 mg/kg for 3 weeks	↑p-Akt ↑ HO-1 ↑ p-FOXO1 ↑ Complex I-V ↑ Electron transport chain activity ↑ APR and ATP	[93]
Adrenocorticotrophic hormone-producing pituitary adenoma	AtT20 cells (mouse corticotrophic tumor cell line)	10, 20, and 40 μM for 24 h	↓ ACTH release ↓ POMC mRNA expression ↓ ACTH protein level ↑ p-JNK/JNK protein level	[94]

↓, Decrease; ↑, Increase.

## Data Availability

The information that supports the findings of this study is available in this article.

## Abbreviations

ursolic acid	UA
mitochondrial membrane permeabilization	MMP
cyclooxygenase 2	COX-2
nuclear Factor Kappa B	NF-κB
mechanistic target of rapamycin	mTOR
chronic lymphocytic leukemia	CLL
reactive oxygen species	ROS
hydrogen peroxide	H2O2
non-small cell lung cancer	NSCLC
non-Hodgkin lymphomas	NHL
tumor necrosis factor	TNF
